# New records of amphibians for Ha Nam Province, Vietnam

**DOI:** 10.3897/BDJ.13.e159973

**Published:** 2025-09-23

**Authors:** Anh Van Pham, Truong Quang Nguyen, Tien Quang Phan, Minh Duc Le, Dung Tien Pham, Cuong The Pham

**Affiliations:** 1 Faculty of Environmental Sciences, University of Science, Vietnam National University, Hanoi, Vietnam Faculty of Environmental Sciences, University of Science, Vietnam National University Hanoi Vietnam; 2 Institute of Biology, Vietnam Academy of Science and Technology, Hanoi, Vietnam Institute of Biology, Vietnam Academy of Science and Technology Hanoi Vietnam; 3 Graduate University of Science and Technology, Vietnam Academy of Science and Technology, Hanoi, Vietnam Graduate University of Science and Technology, Vietnam Academy of Science and Technology Hanoi Vietnam; 4 Central Institute for Natural Resources and Environmental Studies, Vietnam National University, Hanoi, Vietnam Central Institute for Natural Resources and Environmental Studies, Vietnam National University Hanoi Vietnam; 5 Department of Herpetology, American Museum of Natural History, New York, United States of America Department of Herpetology, American Museum of Natural History New York United States of America; 6 Vietnam Environment Sustainable Development Institute, Hanoi, Vietnam Vietnam Environment Sustainable Development Institute Hanoi Vietnam

**Keywords:** distribution, frogs, morphology, taxonomy, Kim Bang Proposed Species and Habitat Conservation Area

## Abstract

**Background:**

The Kim Bang Proposed Species and Habitat Conservation Area (SHCA) is located in Ha Nam Province, northern Vietnam. The terrain of the proposed SHCA is characterised by limestone karst formations and narrow valleys. However, the amphibia fauna of Kim Bang SHCA as well as of Ha Nam Province is poorly studied with only nine recorded species of amphibians from this Province so far.

**New information:**

As a result of our field survey in April 2025, a total of 19 species of amphibians were recorded from Kim Bang SHCA. Ten of them are recorded for the first time from Ha Nam Province, comprising five species of Microhylidae (*Microhyla butleri*, *M. heymonsi*, *M. mukhlesuri*, *M. pulchra* and *Micryletta hekouensis*), one species of Dicroglossidae (*Occidozyga lingnanica*), two species of Ranidae (*Hylarana annamitica*, *Rana johnsi*) and two species of Rhacophoridae (*Kurixalus bisacculus*, *Theloderma lateriticum*). In addition, we provide morphological data and ecological notes of the aforementioned species.

## Introduction

According to Decision No. 1352/TTg of the Prime Minister (2024), the Kim Bang Proposed Species and Habitat Conservation Area, located in Ha Nam Province of Vietnam, was recently included in the National Biodiversity Conservation Planning for the period 2021-2030 with an area of 3,400 hectares (The Government of Vietnam 2024). The topography of the Kim Bang SHCA is characterised by limestone karst formation and narrow valleys (The People's Committee of Ha Nam Province 2018).

In Vietnam, a total of 298 species of amphibians are known (Frost 2025); however, approximately 28% (80 species) of the total recorded species from the country are listed in the IUCN Red List as threatened species (IUCN 2025). In terms of the amphibian fauna, Ha Nam Province is one of the most poorly-studied provinces in Vietnam. In their herpetofaunal list of Vietnam, Nguyen et al. (2009) recorded five species of amphibians from Ha Nam Province. Do et al. (2022) recorded four additional species of amphibians from this Province.

Based on our recent fieldwork in Kim Bang SHCA in 2025, we herein report ten new provincial records of amphibians for Ha Nam Province. The new findings provide critical evidence to support conservation planning and habitat management efforts within the proposed conservation area.

## Materials and methods

**Sampling**

**Figure 1.**
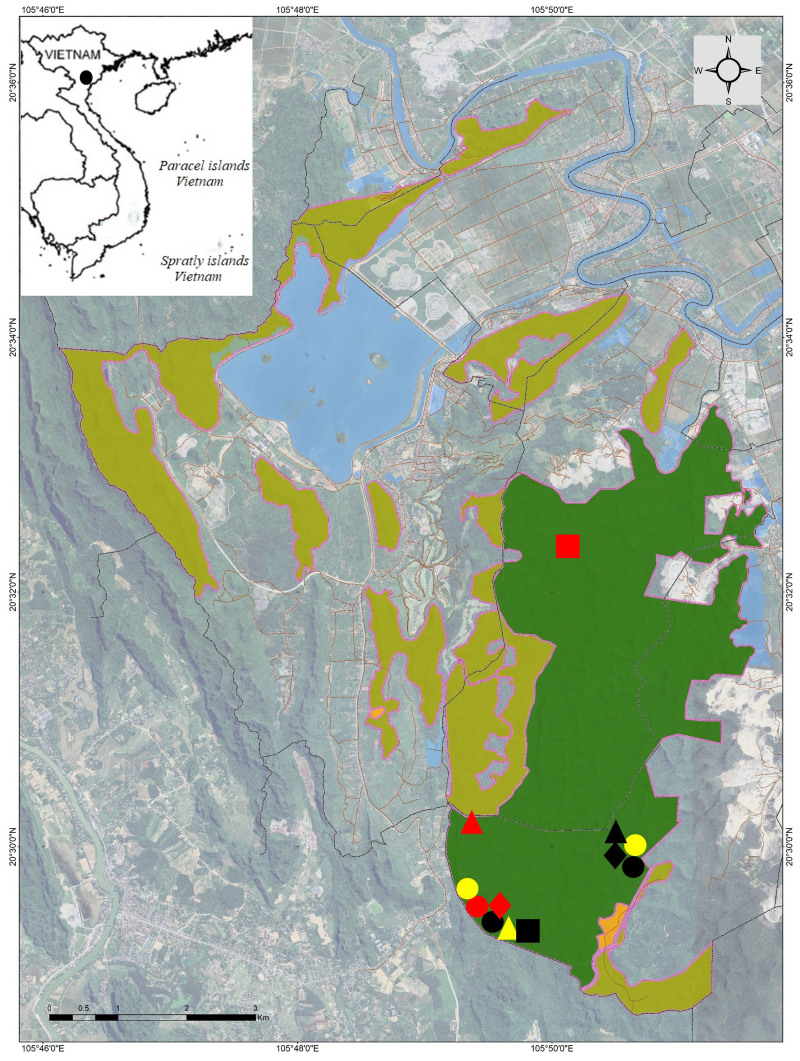
Map showing sampling sites within Kim Bang Species and Habitat Conservation Area in Ha Nam Province, Vietnam. Red circle = *Microhyla butleri*, Black circle = *M. heymonsi*, Yellow circle = *M. mukhlesuri*, Black diamond = *M. pulchra*, Red diamond = *Micryletta hekouensis*, Black triangle = *Occidozyga lingnanica*, Red triangle = *Hylarana annamitica*, Yellow triangle = *Rana johnsi*, Black square = *Kurixalus bisacculus*, Red square = *Theloderma lateriticum*.

A field survey was conducted in Kim Bang SHCA, Ha Nam Province, Vietnam from 21 to 28 April 2025 by Nguyen QT, Pham TC, Phan QT and Pham VA. The coordinates (WGS 84) and elevations were determined by using the GPS Garmin 62SX (Fig. 1).

Specimens were collected by hand between 19:00 h and 22:00 h. After taking photographs in life, specimens were anaesthetised and euthanised in a closed vessel with a piece of cotton wool containing ethyl acetate (Simmons 2002), fixed in 80% ethanol for five hours and then transferred to 70% ethanol for permanent storage. Voucher specimens were subsequently deposited in the collections of the Institute of Biology (IB), Hanoi, Vietnam.

**Morphological characters**

Measurements were taken with a digital caliper to the nearest 0.1 mm. Abbreviations are as follows: (SVL) snout-vent length; (HL) head length, from posterior corner of mandible to tip of snout; (HW) maximum head width, at the angle of jaws; (RL) rostral length, from anterior corner of orbit to tip of snout; (IND) internarial distance; NS: distance from anterior edge of nostril to tip of snout; (EN) distance from anterior corner of eye to posterior edge of nostril; (ED) eye length, from anterior corner to posterior corner of eye; (IOD) minimum distance between upper eyelids; (UEW) maximum width of upper eyelid; (TD) maximum tympanum diameter; (TYE) tympanum-eye distance, from anterior margin of tympanum to posterior corner of the eye; (HAL) hand length, from elbow to tip of third finger; (FL) thigh length, from vent to knee; (TbL) shank length. For webbing formula, we followed Glaw and Vences (2007). Sex was determined by the presence of nuptial pads and based on gonadal inspection.

## Taxon treatments

### Microhyla butleri

Boulenger, 1900

C71105D0-2468-526F-9A88-1BD04FD48F80

#### Materials

- **Type status:** Other material. **Occurrence:** catalogNumber: IB A.6390; individualCount: 1; sex: male; lifeStage: adult; occurrenceID: DE429952-0943-5A9D-BCA1-CFD0B7A7E8D4; **Taxon:** scientificNameID: *Microhyla butleri*; scientificName: *Microhyla butleri*; class: Amphibia; order: Anura; family: Microhylidae; genus: Microhyla; specificEpithet: *butleri*; scientificNameAuthorship: Boulenger, 1900; **Location:** country: Vietnam; countryCode: VN; stateProvince: Ha Nam; county: Kim Bang; municipality: Kim Bang; locality: Kim Bang SHCA; verbatimElevation: 29 m; verbatimLatitude: 20°47.882'N; verbatimLongitude: 105°82.237'E; verbatimCoordinateSystem: WGS84; **Event:** eventDate: 25 April 2025; eventRemarks: collected by Nguyen QT, Pham TC, Phan QT, and Pham VA; **Record Level:** language: en; collectionCode: Amphibians; basisOfRecord: PreservedSpecimen
- **Type status:** Other material. **Occurrence:** catalogNumber: IB A.6391; individualCount: 1; sex: female; lifeStage: adult; occurrenceID: 90C68A34-8714-5B24-BC3C-D4423AC1EA71; **Taxon:** scientificNameID: *Microhyla butleri*; scientificName: *Microhyla butleri*; class: Amphibia; order: Anura; family: Microhylidae; genus: Microhyla; specificEpithet: *butleri*; scientificNameAuthorship: Boulenger, 1901; **Location:** country: Vietnam; countryCode: VN; stateProvince: Ha Nam; county: Kim Bang; municipality: Kim Bang; locality: Kim Bang SHCA; verbatimElevation: 29 m; verbatimLatitude: 20°47.882'N; verbatimLongitude: 105°82.237'E; verbatimCoordinateSystem: WGS84; **Event:** eventDate: 25 April 2025; eventRemarks: collected by Nguyen QT, Pham TC, Phan QT, and Pham VA; **Record Level:** language: en; collectionCode: Amphibians; basisOfRecord: PreservedSpecimen

#### Description

**Figure 2.**
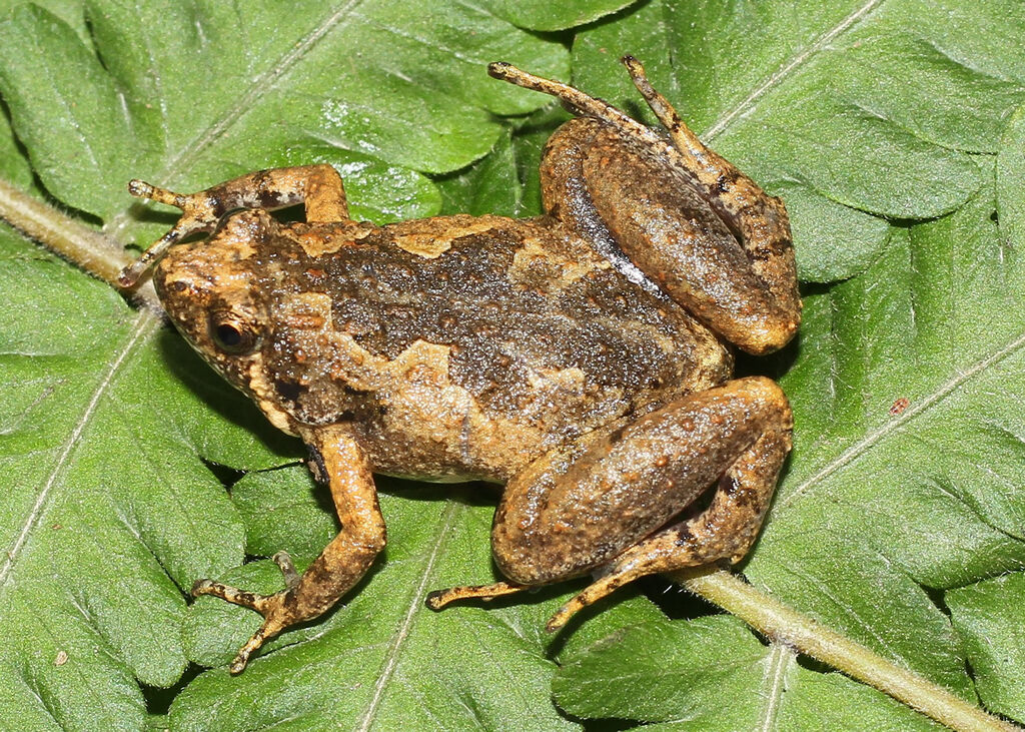
*Microhyla butleri* (adult male, IB A.6390).

**Figure 3.**
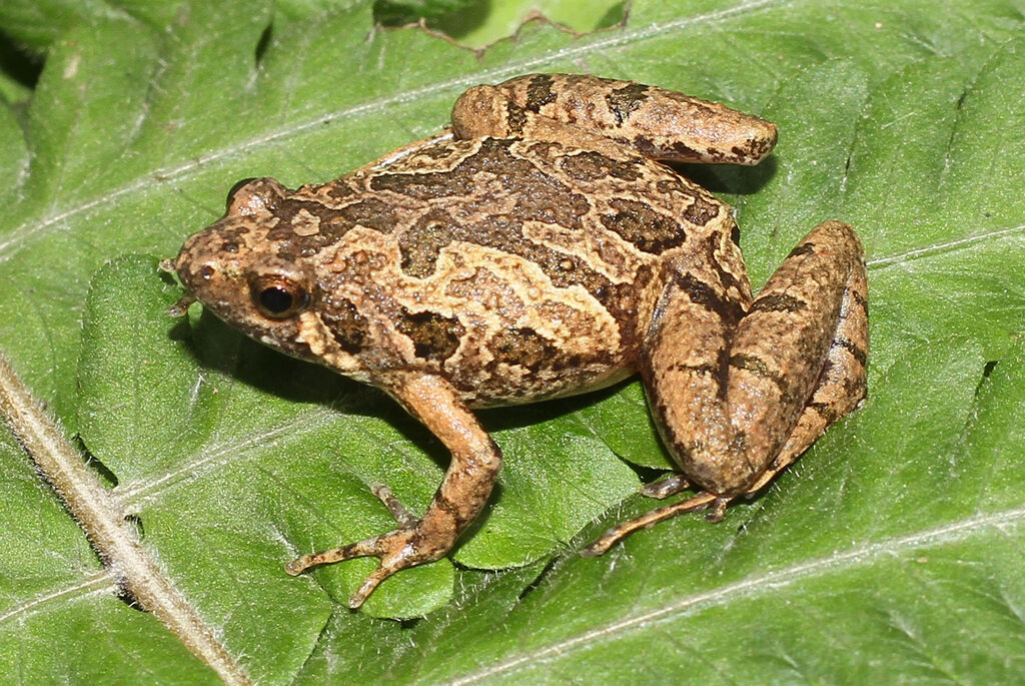
*Microhyla butleri* (adult female, IB A.6391).

Morphological characters of the specimens from Ha Nam Province agreed well with the descriptions of Bourret (1942), Manthey and Grossmann (1997), Bain and Nguyen (2004) and Hecht et al. (2013): SVL 22.1 mm in male (n = 1) and 26.8 mm in female (n = 1); snout round, pronounced, longer than eye (RL 2.9 mm, ED 2.5 mm in male; RL 3.7 mm, ED 2.8 mm in female); interorbital distance 1.6–1.9 times broader than upper eyelid (UEW 1.6 mm, IOD 2.5 mm in male; UEW 1.7 mm, IOD 3.2 mm in female); tympanum invisible; vomerine teeth absent; tongue roundly spatulate and free at the rear half of its length; external vocal sac present in males. Fore-limb slender (HAL 10.4 mm in male and 10.9 mm in female); fingers free of webbing, with slightly developed discs. Hind-limb slender, tibia longer than thigh (TbL 13.4 mm, FL 11.3 mm in male; TbL 14.0 mm, FL 12.5 mm in female); toes with small discs, basally webbed; subarticular tubercle prominent; inner and outer metatarsal tubercles present; tibio-tarsal articulation reaching between eye and tip of snout. Dorsal skin with tiny tubercles on the dorsum and tibia; ventral skin smooth, cloacal region granular. Colouration in life: dorsal surface of head grey, dorsal surface of body brown; a grey stripe extending from eye to shoulder; limbs with dark transverse bars; ventral surface whitish (Figs 2, 3).

#### Distribution

In Vietnam, this species is known from Lao Cai and Ha Giang Provinces in the north, southwards to Dong Nai Province and Ho Chi Minh City (Nguyen et al. 2009, Hecht et al. 2013). Elsewhere, this species is known from China, Myanmar, Laos, Thailand, Cambodia, Indonesia, Malaysia and Singapore (Frost 2025).

#### Ecology

Specimens of *Microhyla butleri* were found on the ground near a small puddle between 20:15 h and 20:45 h. Males were recorded calling at the site and tadpoles were detected in temporary water puddles. The surrounding habitat was a mixed secondary forest of small hardwood and shrubs.

#### Conservation

Conservation status. LC (Least Concern) (IUCN 2025).

### Microhyla heymonsi

Vogt, 1911

27FD4D7E-EFA8-5807-836A-519E612663D1

#### Materials

- **Type status:** Other material. **Occurrence:** catalogNumber: IB A.6392; individualCount: 1; sex: male; lifeStage: adult; occurrenceID: 140A035D-F5C3-5242-8D02-16207B6AC845; **Taxon:** scientificNameID: *Microhyla heymonsi*; scientificName: *Microhyla heymonsi*; class: Amphibia; order: Anura; family: Microhylidae; genus: Microhyla; specificEpithet: *heymonsi*; scientificNameAuthorship: Vogt, 1911; **Location:** country: Vietnam; countryCode: VN; stateProvince: Ha Nam; county: Kim Bang; municipality: Kim Bang; locality: Kim Bang SHCA; verbatimElevation: 25 m; verbatimLatitude: 20°47.689'N; verbatimLongitude: 105°92.435'E; verbatimCoordinateSystem: WGS84; **Event:** eventDate: 24 April 2025; eventRemarks: collected by Nguyen QT, Pham TC, Phan QT, and Pham VA; **Record Level:** language: en; collectionCode: Amphibians; basisOfRecord: PreservedSpecimen
- **Type status:** Other material. **Occurrence:** catalogNumber: IB A.6393; individualCount: 1; sex: female; lifeStage: adult; occurrenceID: D8B5F8D6-412A-51B3-A392-6ED808407436; **Taxon:** scientificNameID: *Microhyla heymonsi*; scientificName: *Microhyla heymonsi*; class: Amphibia; order: Anura; family: Microhylidae; genus: Microhyla; specificEpithet: *heymonsi*; scientificNameAuthorship: Vogt, 1911; **Location:** country: Vietnam; countryCode: VN; stateProvince: Ha Nam; county: Kim Bang; municipality: Kim Bang; locality: Kim Bang SHCA; verbatimElevation: 29 m; verbatimLatitude: 20°47.882'N; verbatimLongitude: 105°82.237'E; verbatimCoordinateSystem: WGS84; **Event:** eventDate: 25 April 2025; eventRemarks: collected by Nguyen QT, Pham TC, Phan QT, and Pham VA; **Record Level:** language: en; collectionCode: Amphibians; basisOfRecord: PreservedSpecimen

#### Description

**Figure 4.**
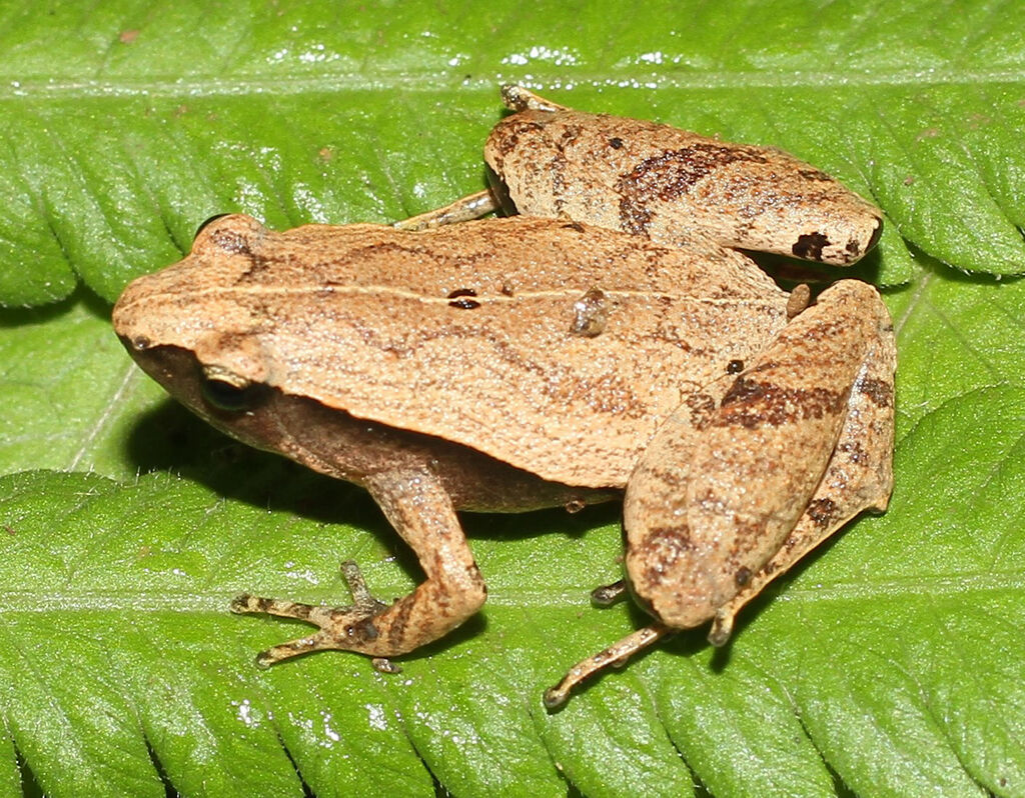
*Microhyla heymonsi* (adult male, IB A.6392).

**Figure 5.**
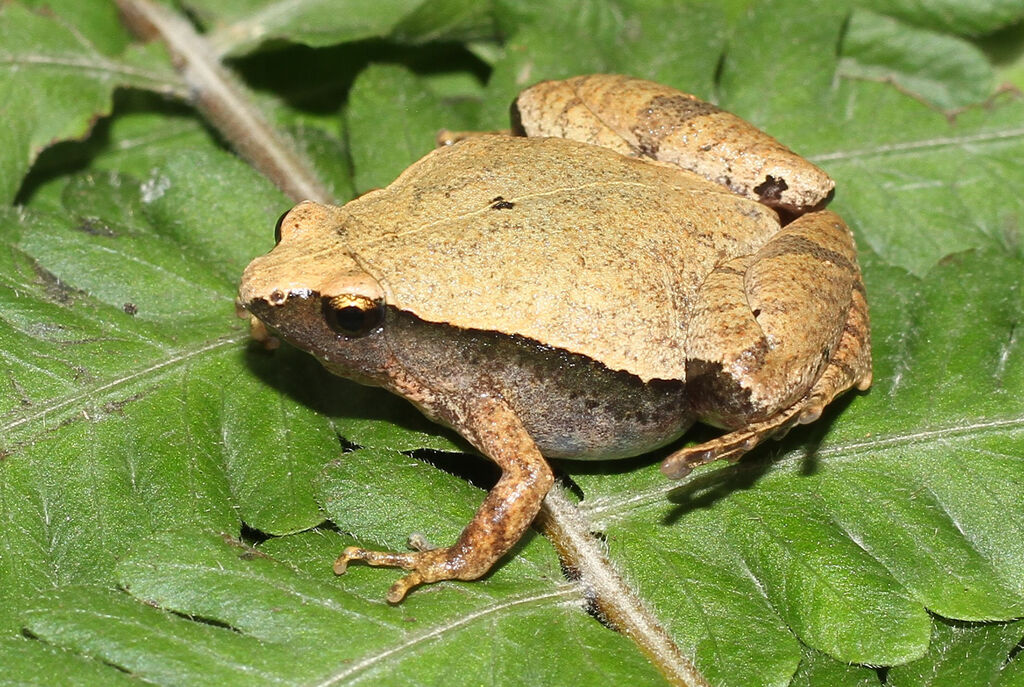
*Microhyla heymonsi* (adult female, IB A.6393).

Morphological characters of the specimens from Ha Nam Province agreed well with the descriptions of Bourret (1942), Manthey and Grossmann (1997), Hecht et al. (2013) and Hoang et al. (2022): SVL 19.5 mm in male (n = 1) and 23.7 mm in female (n = 1); snout profile obtusely pointed, somewhat longer than eye (RL 3.0 mm, ED 2.5 mm in male; RL 3.7 mm, ED 2.2 mm in female); interorbital distance 1.6–1.9 times broader than upper eyelid (UEW 1.3 mm, IOD 2.5 mm in male; UEW 1.8 mm, IOD 2.8 mm in female); tympanum invisible; vomerine teeth absent; tongue roundly spatulate and free at the rear half of its length; external vocal sac present in males. Fore-limb slender (HAL 8.5 mm in male and 9.8 mm in female); fingers free of webbing and tips not swollen. Hind-limb slender, tibia longer than thigh (TbL 11.5 mm, FL 9.9 mm in male; TbL 13.2 mm, FL 11.9 mm in female); toe tips round, not swollen, webbed at base; subarticular tubercle present; inner and outer metatarsal tubercles present; tibio-tarsal articulation reaching tip of snout. Dorsal skin smooth, dorsolateral edges not sharp; ventral skin smooth. Colouration in life: Dorsal surface of head and body pale grey, with a white stripe from tip of snout to cloaca and a small dark spot in the centre of the back; lateral side of head and flank dark brown to black; anterior part of thighs, cloacal region and lower parts of feet black; limbs with thin transverse bars; ventral surface white to grey (Figs 4, 5).

#### Distribution

In Vietnam, this species is known from Lao Cai and Ha Giang Provinces in the north, southwards to Kien Giang and Ca Mau Provinces (Nguyen et al. 2009, Hecht et al. 2013). Elsewhere, this species is known from China, Cambodia, Laos, Myanmar, Thailand and Malaysia (Frost 2025).

#### Ecology

Specimens of *Microhyla heymonsi* were found on the ground near a small puddle or near small streams between 20:00 h and 21:30 h. Males were recorded calling at the site and tadpoles were observed in temporary water puddles. The surrounding habitat was a mixed secondary forest of small hardwood and shrubs.

#### Conservation

Conservation status. LC (Least Concern) (IUCN 2025).

### Microhyla mukhlesuri

Hasan, Islam, Kuramoto, Kurabayashi & Sumida, 2014

76E05299-039A-56E0-9D28-0A174B90CCEB

#### Materials

- **Type status:** Other material. **Occurrence:** catalogNumber: IB A.6394; individualCount: 1; sex: male; lifeStage: adult; occurrenceID: 1E3FB713-F539-515C-A92F-65B6F4B2AB0F; **Taxon:** scientificNameID: *Microhyla mukhlesuri*; scientificName: *Microhyla mukhlesuri*; class: Amphibia; order: Anura; family: Microhylidae; genus: Microhyla; specificEpithet: *mukhlesuri*; scientificNameAuthorship: Hasan, Islam, Kuramoto, Kurabayashi & Sumida, 2014; **Location:** country: Vietnam; countryCode: VN; stateProvince: Ha Nam; county: Kim Bang; municipality: Kim Bang; locality: Kim Bang SHCA; verbatimElevation: 25 m; verbatimLatitude: 20°47.689'N; verbatimLongitude: 105°92.435'E; verbatimCoordinateSystem: WGS84; **Event:** eventDate: 24 April 2025; eventRemarks: collected by Nguyen QT, Pham TC, Phan QT, and Pham VA; **Record Level:** language: en; collectionCode: Amphibians; basisOfRecord: PreservedSpecimen
- **Type status:** Other material. **Occurrence:** catalogNumber: IB A.6395; individualCount: 1; sex: female; lifeStage: adult; occurrenceID: C4811F5E-FE66-5EEA-94F4-B03CEBEB09CB; **Taxon:** scientificNameID: *Microhyla mukhlesuri*; scientificName: *Microhyla mukhlesuri*; class: Amphibia; order: Anura; family: Microhylidae; genus: Microhyla; specificEpithet: *mukhlesuri*; scientificNameAuthorship: Hasan, Islam, Kuramoto, Kurabayashi & Sumida, 2014; **Location:** country: Vietnam; countryCode: VN; stateProvince: Ha Nam; county: Kim Bang; municipality: Kim Bang; locality: Kim Bang SHCA; verbatimElevation: 29 m; verbatimLatitude: 20°47.882'N; verbatimLongitude: 105°82.237'E; verbatimCoordinateSystem: WGS84; **Event:** eventDate: 25 April 2025; eventRemarks: collected by Nguyen QT, Pham TC, Phan QT, and Pham VA; **Record Level:** language: en; collectionCode: Amphibians; basisOfRecord: PreservedSpecimen

#### Description

**Figure 6.**
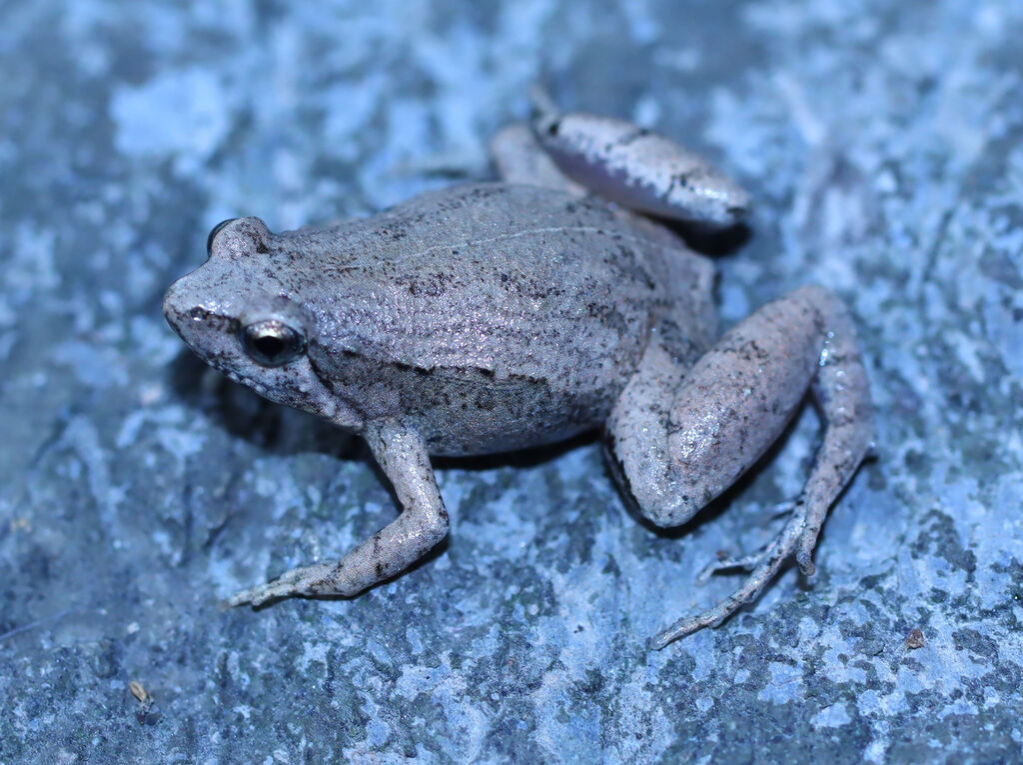
*Microhyla mukhlesuri* (adult male, IB A.6394).

**Figure 7.**
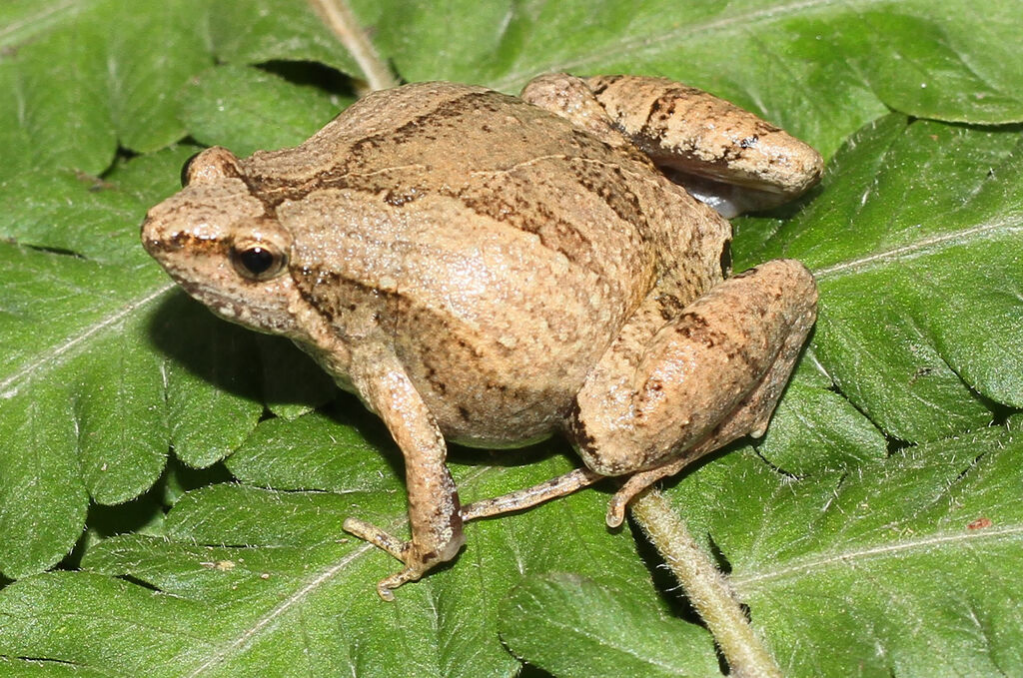
*Microhyla mukhlesuri* (adult female, IB A.6395).

Morphological characters of the specimens from Ha Nam Province agreed well with the description of Hasan et al. (2014): SVL 21.8 mm in male (n = 1) and 26.4 mm in female (n = 1); snout round, longer than eye (RL 3.0 mm, ED 2.0 mm in male; RL 3.6 mm, ED 2.5 mm in female); interorbital distance 1.5–1.8 times broader than upper eyelid (UEW 1.4 mm, IOD 2.5 mm in male; UEW 2.0 mm, IOD 3.0 mm in female); tympanum invisible; vomerine teeth absent; tongue roundly spatulate and free at the rear half of its length; external vocal sac present in males. Fore-limb slender (HAL 8.0 mm in male and 9.4 mm in female); fingers free of webbing and tips not swollen. Hind-limb slender, tibia longer than thigh (TbL 12.0 mm, FL 9.5 mm in male; TbL 12.1 mm, FL 10.0 mm in female); toe tips round, not swollen, webbed at base; subarticular tubercle present; inner and outer metatarsal tubercles present; tibio-tarsal articulation reaching behind the eye. Dorsal skin smooth, dorsolateral ridges discontinuous; ventral skin smooth. Colouration in life: dorsal surface of head and body grey with a dark X-shaped mark on the dorsum, arising from the eyes to the groin; limbs with dark transverse bars; ventral surface whitish, throat and chest mottled with dark brown (Figs 6, 7).

#### Distribution

In Vietnam, this species is known from Lao Cai and Dien Bien Provinces in the north, southwards to Quang Nam Province (Frost 2025). Elsewhere, this species is known from China, Myanmar, Laos, Cambodia, Thailand, India, Bangladesh and Malaysia (Frost 2025).

#### Ecology

Specimens of *Microhyla mukhlesuri* were found on the ground near a small puddle between 20:30 h and 21:00 h. Males were recorded calling at the site and tadpoles were detected in temporary water puddles. The surrounding habitat was a mixed secondary forest of small hardwood and shrubs.

#### Conservation

Conservation status. LC (Least Concern) (IUCN 2025).

### Microhyla pulchra

(Hallowell, 1861)

B482444E-2432-5876-B537-E5B51A9379A7

#### Materials

- **Type status:** Other material. **Occurrence:** catalogNumber: IB A.6396; individualCount: 1; sex: male; lifeStage: adult; occurrenceID: 0C90C684-C3BA-545D-8BF4-28C63E1DB6E7; **Taxon:** scientificNameID: *Microhyla pulchra*; scientificName: *Microhyla pulchra*; class: Amphibia; order: Anura; family: Microhylidae; genus: Microhyla; specificEpithet: *pulchra*; scientificNameAuthorship: (Hallowell, 1861); **Location:** country: Vietnam; countryCode: VN; stateProvince: Ha Nam; county: Kim Bang; municipality: Kim Bang; locality: Kim Bang SHCA; verbatimElevation: 38 m; verbatimLatitude: 20°48.274'N; verbatimLongitude: 105°84.089'E; verbatimCoordinateSystem: WGS84; **Event:** eventDate: 22 April 2025; eventRemarks: collected by Nguyen QT, Pham TC, Phan QT, and Pham VA; **Record Level:** language: en; collectionCode: Amphibians; basisOfRecord: PreservedSpecimen
- **Type status:** Other material. **Occurrence:** catalogNumber: IB A.6397; individualCount: 1; sex: female; lifeStage: adult; occurrenceID: 33396D65-5899-5BBF-A9BA-15BF82EFED83; **Taxon:** scientificNameID: *Microhyla pulchra*; scientificName: *Microhyla pulchra*; class: Amphibia; order: Anura; family: Microhylidae; genus: Microhyla; specificEpithet: *pulchra*; scientificNameAuthorship: (Hallowell, 1861); **Location:** country: Vietnam; countryCode: VN; stateProvince: Ha Nam; county: Kim Bang; municipality: Kim Bang; locality: Kim Bang SHCA; verbatimElevation: 38 m; verbatimLatitude: 20°48.274'N; verbatimLongitude: 105°84.089'E; verbatimCoordinateSystem: WGS84; **Event:** eventDate: 22 April 2025; eventRemarks: collected by Nguyen QT, Pham TC, Phan QT, and Pham VA; **Record Level:** language: en; collectionCode: Amphibians; basisOfRecord: PreservedSpecimen

#### Description

**Figure 8.**
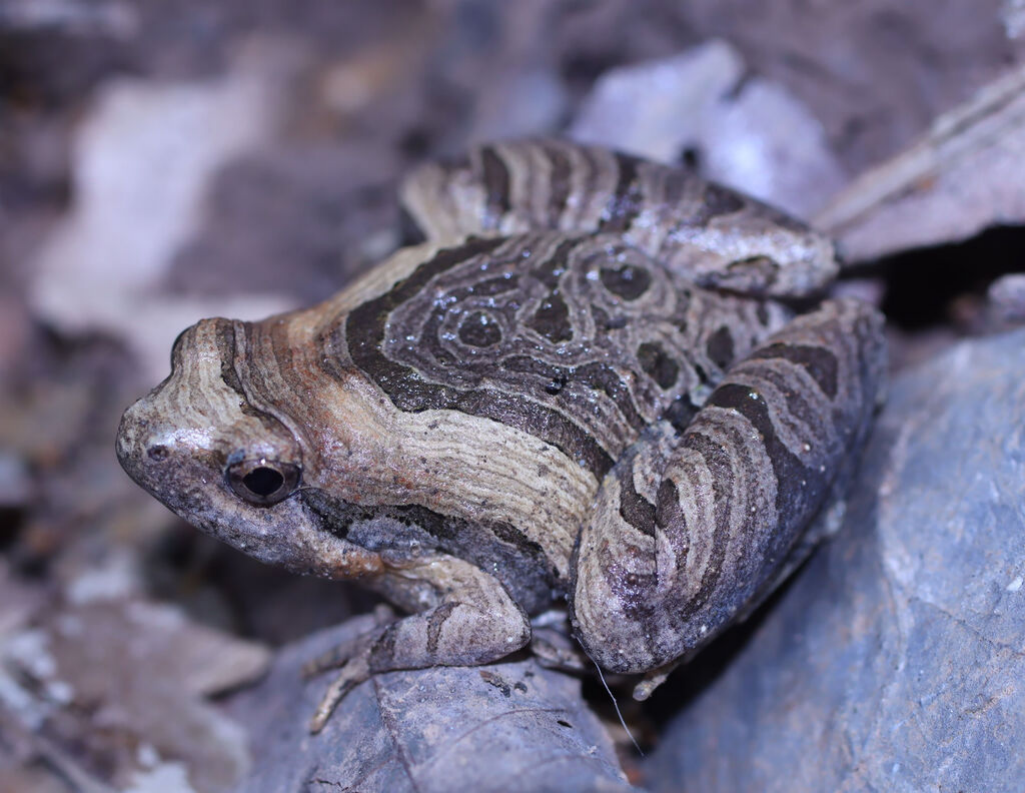
*Microhyla pulchra* (adult male, IB A.6396).

**Figure 9.**
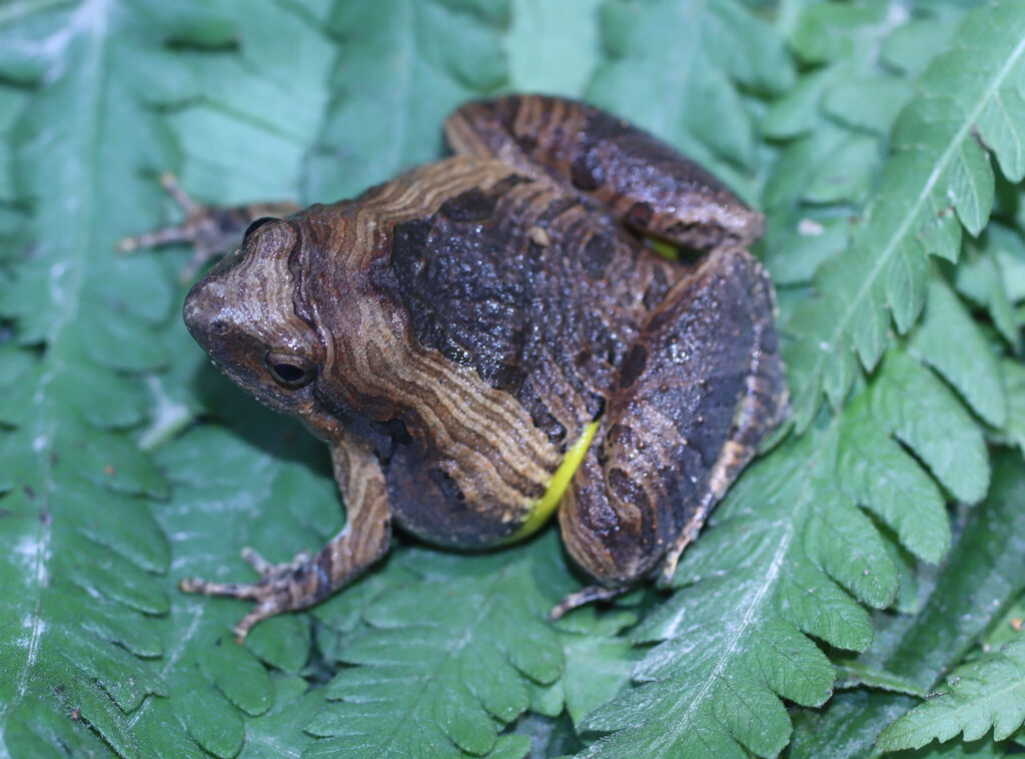
*Microhyla pulchra* (adult female, IB A.6397).

Morphological characters of the specimens from Ha Nam Province agreed well with the descriptions of Bourret (1942), Taylor (1962) and Manthey and Grossmann (1997): SVL 29.2 mm in male (n = 1) and 37.1 mm in female (n = 1); snout obtusely point, slightly pronounced, longer than eye (RL 4.2 mm, ED 3.4 mm in male; RL 5.2 mm, ED 3.8 mm in female); interorbital distance 1.9–2.2 times broader than upper eyelid (UEW 1.6 mm, IOD 3.5 mm in male; UEW 2.4 mm, IOD 4.5 mm in female); tympanum invisible; vomerine teeth absent; tongue roundly spatulate and free at the rear half of its length; external vocal sac present in males. Fore-limb slender (HAL 11.8 mm in male and 13.2 mm in female); fingers free of webbing and tips not swollen. Hind-limb slender, tibia longer than thigh (TbL 18.5 mm, FL 15.6 mm in male; TbL 21.5 mm, FL 17.6 mm in female); toe tips round, not swollen, toes almost 1/2 webbed, webbing formula: I1/2–2II1–3III2–3½IV31/3–2V; subarticular tubercle present; inner and outer metatarsal tubercles present; tibio-tarsal articulation reaching tip of snout. Dorsal skin with a few scattered small tubercles; ventral skin smooth; cloacal region granular. Colouration in life: dorsum light brown, with a dark brown Λ-shaped pattern on the back, containing several dark and light lines, outer part bordered by several light lines; canthus rostralis and flanks dark brown; limbs with transverse bars; groin and anterior part of thigh yellow; ventral surface whitish yellow, chin and throat with black marbling (Figs 8, 9).

#### Distribution

In Vietnam, this species is known from Lao Cai and Ha Giang Provinces in the north, southwards to Lam Dong and Dong Nai Provinces (Nguyen et al. 2009, Hecht et al. 2013). Elsewhere, this species is known from China, Myanmar, Laos, Thailand, Cambodia, Malaysia and Singapore (Frost 2025).

#### Ecology

Specimens of *Microhyla pulchra* were found on the ground near a small puddle or near a pond between 20:00 h and 21:30 h. Males were recorded calling at the site and tadpoles were detected in temporary water puddles. The surrounding habitat was a mixed secondary forest of small hardwood and shrubs.

#### Conservation

Conservation status. LC (Least Concern) (IUCN 2025).

### Micryletta hekouensis

Liu, Hou, Mo & Rao, 2021

7F613987-5ED7-5335-8358-B46AE49F06C1

#### Materials

- **Type status:** Other material. **Occurrence:** catalogNumber: IB A.6398; individualCount: 1; sex: male; lifeStage: adult; occurrenceID: 9B505AB8-0DE7-56F1-852F-C9244A81B4EF; **Taxon:** scientificNameID: *Micryletta hekouensis*; scientificName: *Micryletta hekouensis*; class: Amphibia; order: Anura; family: Microhylidae; genus: Micryletta; specificEpithet: *hekouensis*; scientificNameAuthorship: Liu, Hou, Mo & Rao, 2021; **Location:** country: Vietnam; countryCode: VN; stateProvince: Ha Nam; county: Kim Bang; municipality: Kim Bang; locality: Kim Bang SHCA; verbatimElevation: 24 m; verbatimLatitude: 20°45.637'N; verbatimLongitude: 105°80.930'E; verbatimCoordinateSystem: WGS84; **Event:** eventDate: 25 April 2025; eventRemarks: collected by Nguyen QT, Pham TC, Phan QT, and Pham VA; **Record Level:** language: en; collectionCode: Amphibians; basisOfRecord: PreservedSpecimen
- **Type status:** Other material. **Occurrence:** catalogNumber: IB A.6399; individualCount: 1; sex: female; lifeStage: adult; occurrenceID: 9101472E-4D42-5867-BEE4-1BE6BB35F512; **Taxon:** scientificNameID: *Micryletta hekouensis*; scientificName: *Micryletta hekouensis*; class: Amphibia; order: Anura; family: Microhylidae; genus: Micryletta; specificEpithet: *hekouensis*; scientificNameAuthorship: Liu, Hou, Mo & Rao, 2021; **Location:** country: Vietnam; countryCode: VN; stateProvince: Ha Nam; county: Kim Bang; municipality: Kim Bang; locality: Kim Bang SHCA; verbatimElevation: 24 m; verbatimLatitude: 20°45.637'N; verbatimLongitude: 105°80.930'E; verbatimCoordinateSystem: WGS84; **Event:** eventDate: 25 April 2025; eventRemarks: collected by Nguyen QT, Pham TC, Phan QT, and Pham VA; **Record Level:** language: en; collectionCode: Amphibians; basisOfRecord: PreservedSpecimen

#### Description

**Figure 10.**
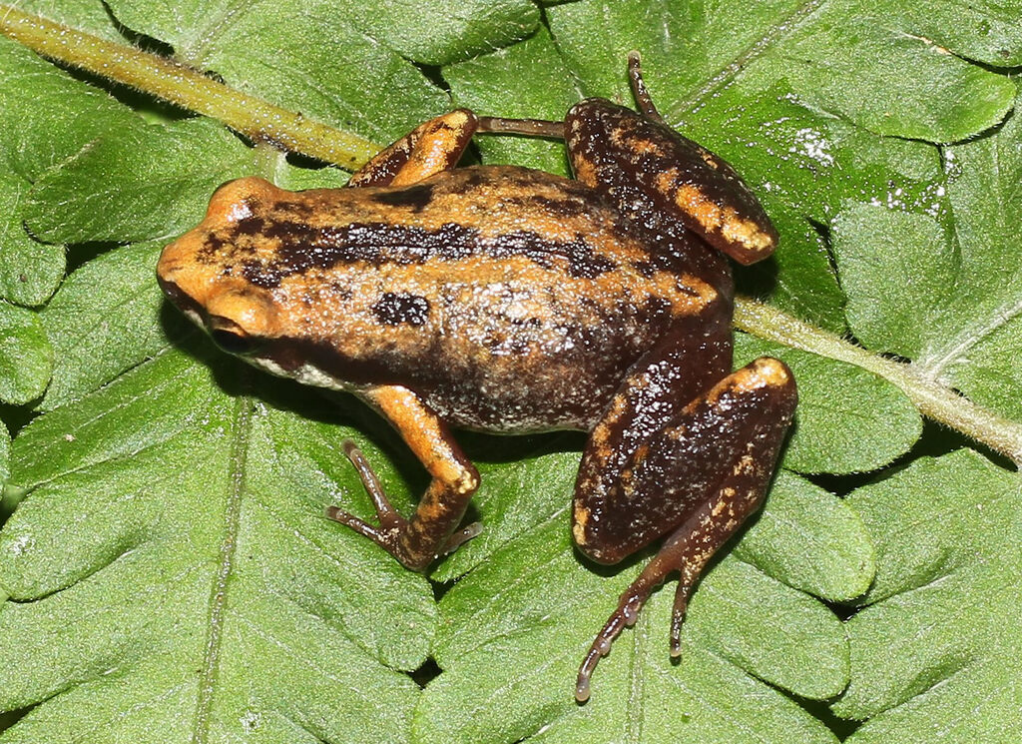
*Micryletta hekouensis* (adult male, IB A.6398).

**Figure 11.**
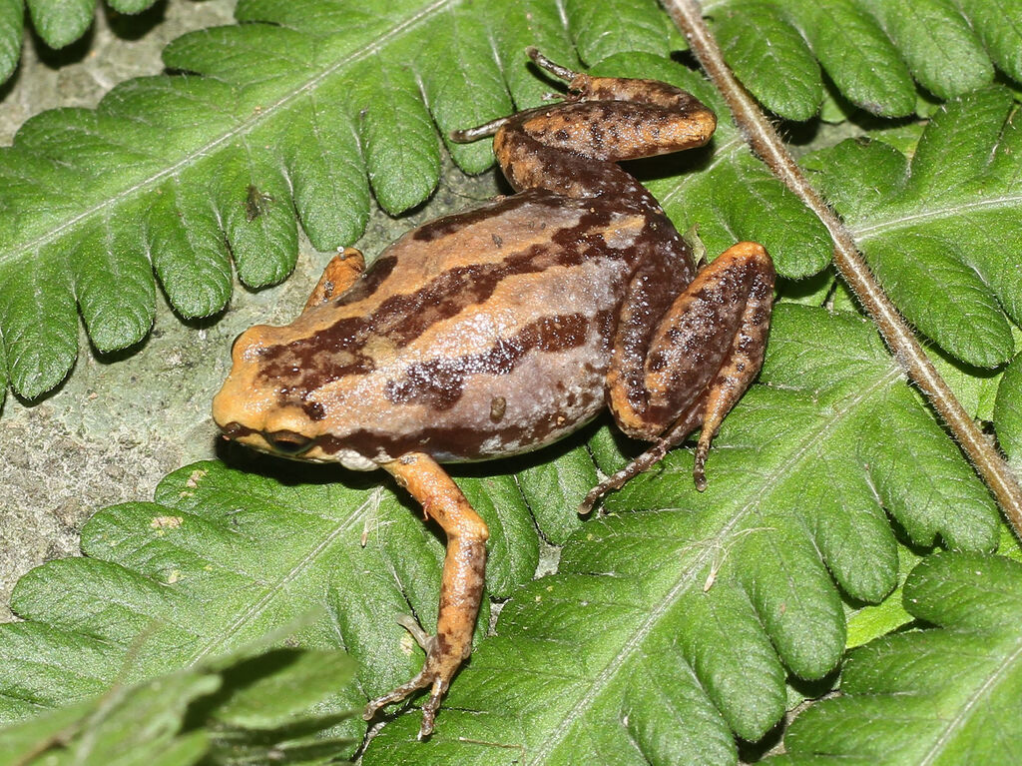
*Micryletta hekouensis* (adult female, IB A.6399).

Morphological characters of the specimens from Ha Nam Province agreed well with the descriptions of Liu et al. (2021) and Nguyen et al. (2024): SVL 22.3 mm in male (n = 1) and 27.5 mm in female (n = 1); head wider than long (HW 6.9 mm, HL 6.2 mm in male; HW 7.8 mm, HL 7.2 mm in female); snout abruptly round in dorsal view and slightly acuminate in profile, longer than eye (RL 3.3 mm, ED 2.5 mm in male; RL 3.9 mm, ED 2.9 mm in female); interorbital distance 1.9–2.3 times broader than upper eyelid (UEW 1.4 mm, IOD 3.2 mm in male; UEW 2.0 mm, IOD 3.8 mm in female); tympanum visible, round (TD 0.6 mm in male, 0.8 mm in female); vomerine teeth absent; tongue oval, with no notch at posterior tip; external vocal sac present in males. Fore-limb slender (HAL 11.9 mm in male and 14.2 mm in female); fingers free of webbing, tips round and not dilated; nuptial pad absent. Hind-limb slender, tibia longer than thigh (TbL 10.9 mm, FL 10.0 mm in male; TbL 12.5 mm, FL 11.8 mm in female); toe tips round and not dilated; webbing between toes absent; subarticular tubercle present; inner and outer metatarsal tubercles present; tibio-tarsal articulation reaching level of front of eye. Dorsal skin smooth, without dorsolateral fold, flanks and hind-limb with small tubercles; ventral skin smooth. Colouration in life: dorsal surface of head and body as well as upper arm golden, back with two indistinct parallel longitudinal grey stripes; lateral sides of head and body black; ventral side of body and limbs pinkish-brown, chin region brownish-black; chest and lateral region of belly with small and irregular white marbling (Figs 10, 11).

#### Distribution

In Vietnam, this species is recently known from Hai Phong City and Ninh Binh Province (Nguyen et al. 2024, Frost 2025). Elsewhere, this species is only known from China (Frost 2025).

#### Ecology

Specimens of *Micryletta hekouensis* were found on the ground near a small stream between 19:30 h and 21:30 h. Males were recorded calling at the site and tadpoles were observed in temporary water puddles. The surrounding habitat was a mixed secondary forest of small hardwood and shrubs.

#### Conservation

Conservation status. Not evaluated (IUCN 2025).

### Occidozyga lingnanica

Lyu & Wang, 2022

92037747-C397-50B2-A90F-12936D482431

#### Materials

- **Type status:** Other material. **Occurrence:** catalogNumber: IB. A.6400; individualCount: 1; sex: male; lifeStage: adult; occurrenceID: 374BC282-71D4-55C0-B309-66CE094CE73D; **Taxon:** scientificNameID: *Occidozyga lingnanica*; scientificName: *Occidozyga lingnanica*; class: Amphibia; order: Anura; family: Dicroglossidae; genus: Occidozyga; specificEpithet: *lingnanica*; scientificNameAuthorship: Lyu & Wang, 2022; **Location:** country: Vietnam; countryCode: VN; stateProvince: Ha Nam; county: Kim Bang; municipality: Kim Bang; locality: Kim Bang SHCA; verbatimElevation: 38 m; verbatimLatitude: 20°48.274'N; verbatimLongitude: 105°84.089'E; verbatimCoordinateSystem: WGS84; **Event:** eventDate: 22 April 2025; eventRemarks: collected by Nguyen QT, Pham TC, Phan QT, and Pham VA; **Record Level:** language: en; collectionCode: Amphibians; basisOfRecord: PreservedSpecimen
- **Type status:** Other material. **Occurrence:** catalogNumber: IB A.6401; individualCount: 1; sex: female; lifeStage: adult; occurrenceID: E2D4B011-7C8B-5287-A48C-E1A05970D2D2; **Taxon:** scientificNameID: *Occidozyga lingnanica*; scientificName: *Occidozyga lingnanica*; class: Amphibia; order: Anura; family: Dicroglossidae; genus: Occidozyga; specificEpithet: *lingnanica*; scientificNameAuthorship: Lyu & Wang, 2022; **Location:** country: Vietnam; countryCode: VN; stateProvince: Ha Nam; county: Kim Bang; municipality: Kim Bang; locality: Kim Bang SHCA; verbatimElevation: 38 m; verbatimLatitude: 20°48.274'N; verbatimLongitude: 105°84.089'E; verbatimCoordinateSystem: WGS84; **Event:** eventDate: 22 April 2025; eventRemarks: collected by Nguyen QT, Pham TC, Phan QT, and Pham VA; **Record Level:** language: en; collectionCode: Amphibians; basisOfRecord: PreservedSpecimen

#### Description

**Figure 12.**
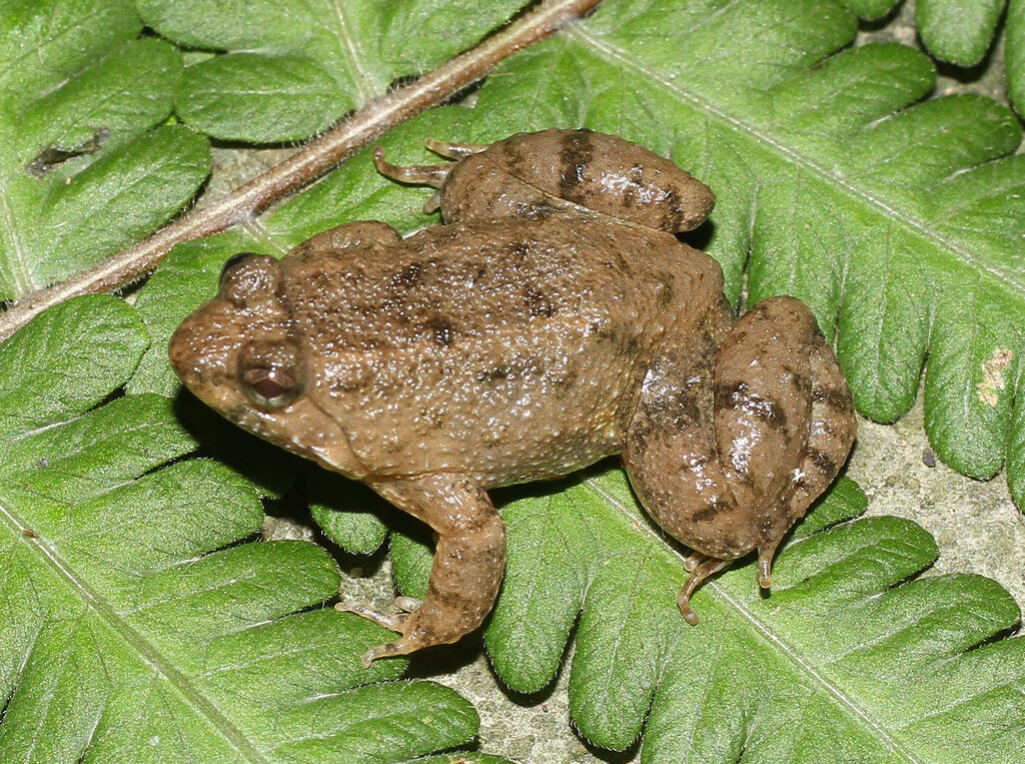
*Occidozyga lingnanica* (adult male, IB A.6400).

**Figure 13.**
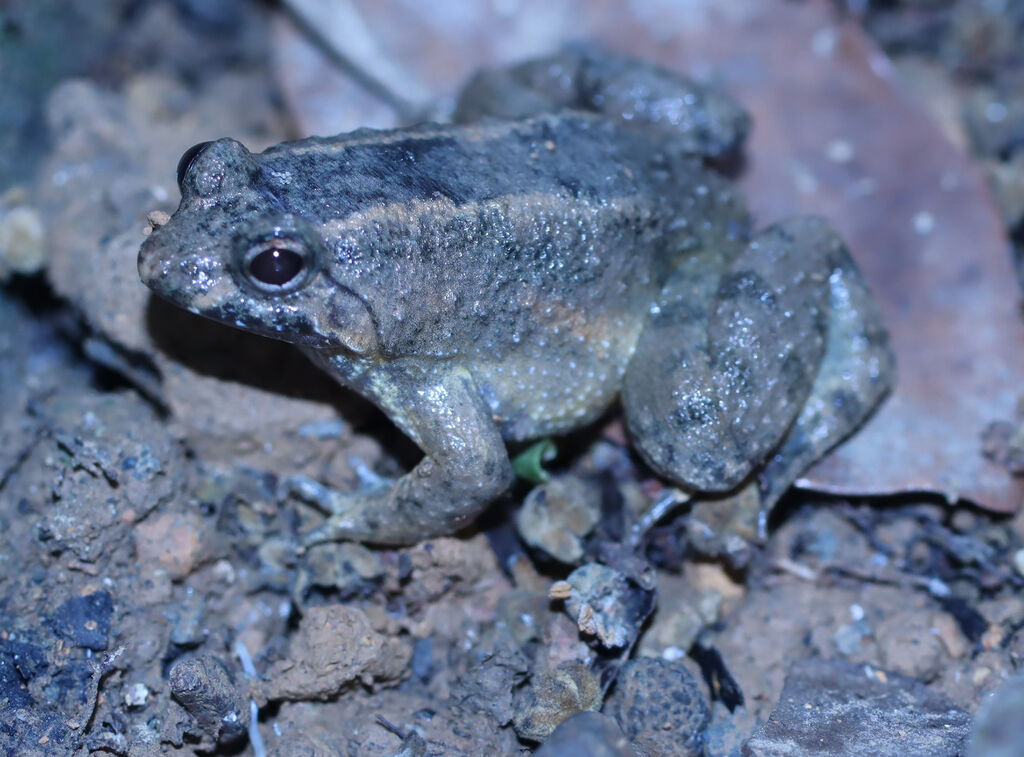
*Occidozyga lingnanica* (adult female, IB A.6401).

Morphological characters of the specimens from Ha Nam Province agreed well with the description of Lyu et al. (2022): SVL 22.2 mm in male (n = 1) and 28.4 mm in female (n = 1); head longer than wide (HW 7.8 mm, HL 8.2 mm in male; HW 8.7 mm, HL 8.9 mm in female); snout round in dorsal view and profile, longer than eye (RL 3.5 mm, ED 2.8 mm in male; RL 4.0 mm, ED 3.0 mm in female); interorbital distance 1.5–2.0 times broader than upper eyelid (UEW 1.5 mm, IOD 3.0 mm in male; UEW 2.1 mm, IOD 3.1 mm in female); tympanum hidden, edge invisible; vomerine teeth absent; tongue round posteriorly; external vocal sac present in males. Fore-limb slender (HAL 8.5 mm in male and 11.3 mm in female); fingers free of webbing, tips round and not dilated; relative finger lengths II = I < IV < III; nuptial pad present in male. Hind-limb slender, tibia longer than thigh (TbL 11.0 mm, FL 10.1 mm in male; TbL 14.0 mm, FL 12.8 mm in female); relative lengths I < II < V < III < IV; toe tips round, dilated into round discs; toes fully webbed; subarticular tubercles present; inner and outer metatarsal tubercles present; tibio-tarsal articulation reaching at the posterior margin of supratympanic fold. Dorsal surface of body and limb rough, with large tubercles, flanks with tubercles; a faint fold across head between orbits; supratympanic fold distinct; dorsolateral fold absent; ventral surface with flat tubercles; a fold across breast; and dense granules on the ventral tarsi. Colouration in life: Dorsal surface greyish-brown with irregular black speckles; a narrow transverse dark bar between orbits; mid-dorsal stripe yellowish-brown, but indistinct; limbs with dark brown transverse bars; throat dark grey with white mottling; chest and belly uniform creamy white (Figs 12, 13).

#### Distribution

In Vietnam, this species is known from Ninh Binh Province (unpublished data). Elsewhere, this species is known from China (Frost 2025).

#### Ecology

Specimens of *Occidozyga lingnanica* were found on the ground near a small stream between 19:00 h and 21:30 h. Males were recorded calling at the site and tadpoles were detected in temporary water puddles. The surrounding habitat was a mixed secondary forest of small hardwood and shrubs.

#### Conservation

Conservation status. Not evaluated (IUCN 2025).

### Hylarana annamitica

(Sheridan & Stuart, 2018)

4DDB41F6-0CD3-526A-B30E-B6831FB2F0DD

#### Materials

- **Type status:** Other material. **Occurrence:** catalogNumber: IB A.6402; individualCount: 1; sex: male; lifeStage: adult; occurrenceID: 64B66777-6AD0-54D9-89BF-C188F8015D34; **Taxon:** scientificNameID: *Hylarana annamitica*; scientificName: *Hylarana annamitica*; class: Amphibia; order: Anura; family: Ranidae; genus: Hylarana; specificEpithet: *annamitica*; scientificNameAuthorship: (Sheridan & Stuart, 2018); **Location:** country: Vietnam; countryCode: VN; stateProvince: Ha Nam; county: Kim Bang; municipality: Kim Bang; locality: Kim Bang SHCA; verbatimElevation: 52 m; verbatimLatitude: 20°52.230'N; verbatimLongitude: 105°81.909'E; verbatimCoordinateSystem: WGS84; **Event:** eventDate: 24 April 2025; eventRemarks: collected by Nguyen QT, Pham TC, Phan QT, and Pham VA; **Record Level:** language: en; collectionCode: Amphibians; basisOfRecord: PreservedSpecimen
- **Type status:** Other material. **Occurrence:** catalogNumber: IB A.6403; individualCount: 1; sex: male; lifeStage: adult; occurrenceID: 39EAD54F-64E2-5C7A-BFDE-6E50ED7EF0CA; **Taxon:** scientificNameID: *Hylarana annamitica*; scientificName: *Hylarana annamitica*; class: Amphibia; order: Anura; family: Ranidae; genus: Hylarana; specificEpithet: *annamitica*; scientificNameAuthorship: (Sheridan & Stuart, 2018); **Location:** country: Vietnam; countryCode: VN; stateProvince: Ha Nam; county: Kim Bang; municipality: Kim Bang; locality: Kim Bang SHCA; verbatimElevation: 52 m; verbatimLatitude: 20°52.230'N; verbatimLongitude: 105°81.909'E; verbatimCoordinateSystem: WGS84; **Event:** eventDate: 24 April 2025; eventRemarks: collected by Nguyen QT, Pham TC, Phan QT, and Pham VA; **Record Level:** language: en; collectionCode: Amphibians; basisOfRecord: PreservedSpecimen

#### Description

**Figure 14.**
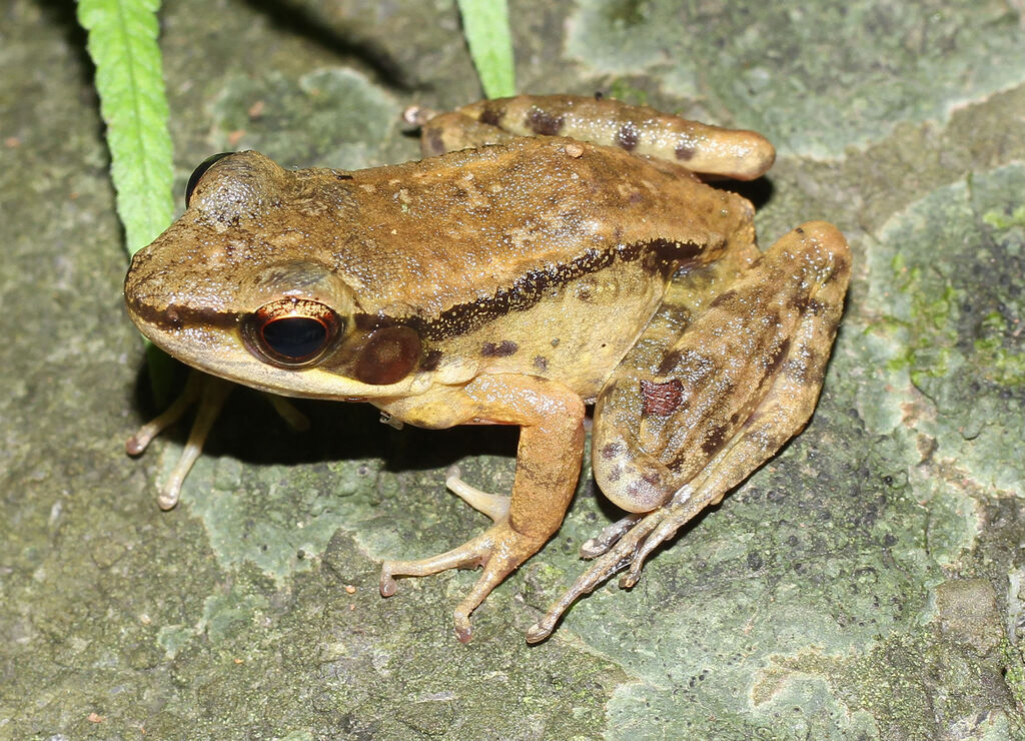
*Hylarana nigrovittata* (adult male, IB A.6402).

Morphological characters of the specimens from Ha Nam Province agreed well with the descriptions of Sheridan and Stuart (2018) and To et al. (2025): SVL 40.1–40.4 mm in males (n = 2); head longer than wide (HW 14.2–14.8 mm, HL 16.5–17.1 mm); snout obtusely pointed in dorsal view, longer than eye (RL 6.9–7.1 mm, ED 6.2–6.5 mm); interorbital distance 1.3–1.5 times broader than upper eyelid (UEW 3.6–3.7 mm, IOD 4.9–5.2 mm); tympanum distinct, round, more than half eye diameter (TD 3.7–3.9 mm); vomerine teeth obliquely angled; tongue notched posteriorly; vocal sac openings near corner of jaw in males. Fore-limb slender (HAL 19.0–20.5 mm); fingers free of webbing, tips of fingers expanded into small discs with circum-marginal grooves, relative finger lengths IV < II < I < III; nuptial pad present in male. Hind-limb slender, tibia longer than thigh (TbL 22.0–24.0 mm; FL 19.0–21.6 mm); toe tips expanded into discs with circum-marginal grooves; webbing present, webbing formula: I1/2–3/4II0–1III0–1IV1–0V inner metatarsal tubercle elongate; tibio-tarsal articulation reaching between eye and nose. Dorsal skin finely granular; supratympanic fold distinct; dorsolateral fold distinct; throat, chest, belly and ventral surface of thigh smooth. Colouration in life: Dorsum reddish-brown, with a few dark brown mottling spots; lip white yellow; dorsal surface of fore-limb and hind-limb with dark crossbars; dorsolateral fold dark brown; flanks pale grey with dark brown spots; ventral surface slightly yellow, dark mottling on throat, less mottling on chest and underside of thighs (Fig. 14).

#### Distribution

In Vietnam, this species is known from Bac Kan, Vinh Phuc, Thanh Hoa, Nghe An, Ha Tinh, Hue and Quang Nam Provinces (Sheridan and Stuart 2018, To et al. 2025). Elsewhere, this species is known from Laos (Frost 2025).

#### Ecology

Specimens of *Hylarana annamitica* were found on the ground near two small streams between 19:00 h and 21:00 h. The surrounding habitat was a secondary forest of small hardwood and shrubs.

#### Conservation

Conservation status. LC (Least Concern) (IUCN 2025).

### Rana johnsi

Smith, 1921

CEF5E4C2-59C0-5BC7-AA89-F53A8F17B804

#### Materials

- **Type status:** Other material. **Occurrence:** catalogNumber: IB A.6404; individualCount: 1; sex: male; lifeStage: adult; occurrenceID: C54EC80E-8EF6-54FF-9BD0-71424797A132; **Taxon:** scientificNameID: *Rana johnsi*; scientificName: *Rana johnsi*; class: Amphibia; order: Anura; family: Ranidae; genus: Rana; specificEpithet: *johnsi*; scientificNameAuthorship: Smith, 1921; **Location:** country: Vietnam; countryCode: VN; stateProvince: Ha Nam; county: Kim Bang; municipality: Kim Bang; locality: Kim Bang SHCA; verbatimElevation: 24 m; verbatimLatitude: 20°45.637'N; verbatimLongitude: 105°80.930'E; verbatimCoordinateSystem: WGS84; **Event:** eventDate: 25 Apri 2025; eventRemarks: collected by Nguyen QT, Pham TC, Phan QT, and Pham VA; **Record Level:** language: en; collectionCode: Amphibians; basisOfRecord: PreservedSpecimen

#### Description

**Figure 15.**
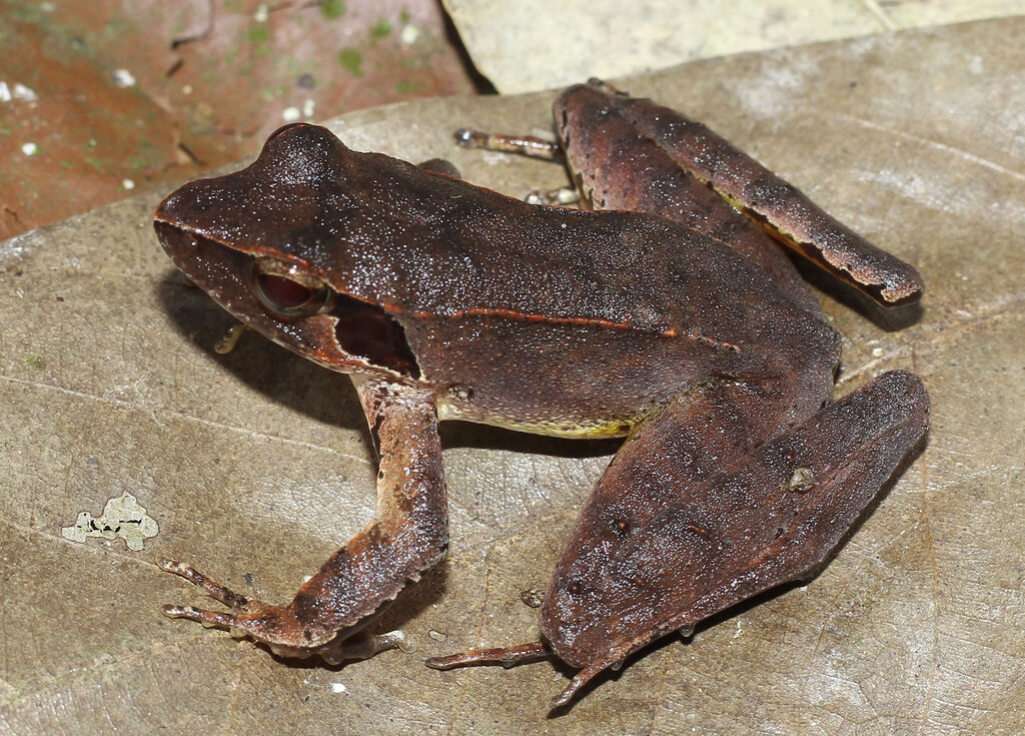
*Rana johnsi* (adult male, IB A.6404).

Morphological characters of the specimen from Ha Nam Province agreed well with the descriptions of Bourret (1942) and Hecht et al. (2013): SVL 40.9 mm in male (n = 1); head longer than wide (HW 12.2 mm, HL 19.0 mm); snout obtusely pointed, pronounced, longer than eye (SL 6.6 mm, ED 5.6 mm); interorbital distance 1.3 times broader than upper eyelid (UEW 3.5 mm, IOD 4.4 mm); tympanum distinct, round, more than half eye diameter (TD 3.6 mm); vomerine teeth present; tongue notched posteriorly; external vocal sac present in males. Fore-limb slender (HAL 20.0 mm); fingers free of webbing, tips of fingers expanded into small discs, relative finger lengths II < I < IV < III; nuptial pad present in male. Hind-limb slender, tibia longer than thigh (TbL 27.2 mm; TbL 24.4 mm); toe tips expanded into discs; webbing present, webbing formula: I1/2–3/4II0–1III0–1IV1 3/4–0V; inner metatarsal tubercle elongate; outer metatarsal tubercle absent; tibio-tarsal articulation reaching beyond tip of snout. Dorsal skin with some small granules; a Λ-shaped fold between shoulders; some short, oblique dermal folds on limbs; supratympanic and dorsolateral folds distinct; throat, chest, belly and ventral surface of thigh smooth. Colouration in life: Dorsum brown; flank light brown; a small black stripe extending from nostril to eye; tympanum covered by a black lozenge; lateral side of limb with dark pattern; ventral surface cream; gular region marbled with white; thigh yellow (Fig. 15).

#### Distribution

In Vietnam, this species is known from Lao Cai and Ha Giang Provinces in the north, southwards to Lam Dong and Dong Nai Provinces (Nguyen et al. 2009). Elsewhere, this species is known from China, Laos, Cambodia and Thailand (Frost 2025).

#### Ecology

Specimen of *Rana johnsi* was found on the ground near a small stream at 21:00 h. The surrounding habitat was a secondary forest of small hardwood and shrubs.

#### Conservation

Conservation status. LC (Least Concern) (IUCN 2025).

### Kurixalus bisacculus

(Taylor 1962)

20A292E5-CCF7-5D26-BEB1-A74F8B9CEF7A

#### Materials

- **Type status:** Other material. **Occurrence:** catalogNumber: IB A.6405; individualCount: 1; sex: male; lifeStage: adult; occurrenceID: A3A09104-3FD4-55DC-9790-B8628405BEFA; **Taxon:** scientificNameID: *Kurixalus bisacculus*; scientificName: *Kurixalus bisacculus*; class: Amphibia; order: Anura; family: Racophoridae; genus: Kurixalus; specificEpithet: *bisacculus*; scientificNameAuthorship: (Taylor, 1962); **Location:** country: Vietnam; countryCode: VN; stateProvince: Ha Nam; county: Kim Bang; municipality: Kim Bang; locality: Kim Bang SHCA; verbatimElevation: 24 m; verbatimLatitude: 20°45.637'N; verbatimLongitude: 105°80.930'E; verbatimCoordinateSystem: WGS84; **Event:** eventDate: 25 April 2025; eventRemarks: collected by Nguyen QT, Pham TC, Phan QT, and Pham VA; **Record Level:** language: en; collectionCode: Amphibians; basisOfRecord: PreservedSpecimen
- **Type status:** Other material. **Occurrence:** catalogNumber: IB A.6406; individualCount: 1; sex: male; lifeStage: adult; occurrenceID: 6602AEDA-2AB4-5166-8E8F-F1F59D071601; **Taxon:** scientificNameID: *Kurixalus bisacculus*; scientificName: *Kurixalus bisacculus*; class: Amphibia; order: Anura; family: Racophoridae; genus: Kurixalus; specificEpithet: *bisacculus*; scientificNameAuthorship: (Taylor, 1962); **Location:** country: Vietnam; countryCode: VN; stateProvince: Ha Nam; county: Kim Bang; municipality: Kim Bang; locality: Kim Bang SHCA; verbatimElevation: 24 m; verbatimLatitude: 20°45.637'N; verbatimLongitude: 105°80.930'E; verbatimCoordinateSystem: WGS84; **Event:** eventDate: 25 April 2025; eventRemarks: collected by Nguyen QT, Pham TC, Phan QT, and Pham VA; **Record Level:** language: en; collectionCode: Amphibians; basisOfRecord: PreservedSpecimen

#### Description

**Figure 16.**
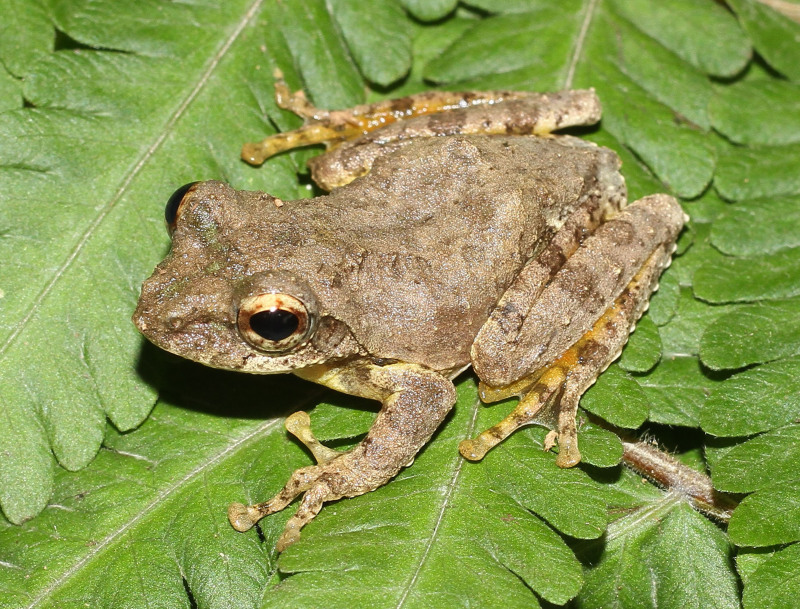
*Kurixalus bisacculus* (adult male, IB A.6405).

Morphological characters of the specimens from Ha Nam Province agreed well with the descriptions of Taylor (1962): SVL 30.5–32.4 mm in males (n = 2); head longer than wide (HW 10.5–11.5 mm, HL 11.3–11.9 mm); snout pointed anteriorly, as long as the eye (RL 5.0 mm, ED 5.0–5.3 mm); interorbital distance 1.2–1.4 times broader than upper eyelid (UEW 2.5–2.9 mm, IOD 3.5–3.7 mm); tympanum distinct, round, less than half eye diameter (TD 2.1–32.2 mm); vomerine teeth present; tongue notched posteriorly; external vocal sac present in males. Fore-limb slender (HAL 14.2–14.5 mm); fingers free of webbing, tips of fingers with enlarged discs; nuptial pad present in males. Hind-limb slender, tibia longer than thigh (TbL 16.0–17.0 mm; FL 14.0–15.6 mm); toe tips expanded into discs; webbing present, webbing formula: I1/2–1II0–1 1/2III0–1IV1–1/2V; inner metatarsal tubercle present; tibio-tarsal articulation reaching the eye. Skin on head, eyelids and occiput, dorsum with scattered tubercles; dermal fringes along outer edges of forearm and tarsus present; chin granular, chest nearly smooth; venter and lower part of flank granular. Colouration in life: Dorsal surface of head and body light brown with green marking, occiput with a dark green marking in triangular shape; chin cream with dark spots; throat, chest, venter and underside of limbs cream (Fig. 16).

#### Diagnosis

In Vietnam, this species is known from Lao Cai Province in the north, southwards to Quang Binh Province (Yu et al. 2017, Frost 2025). Elsewhere, the species has been reported from Cambodia, China, Laos, Myanmar and Thailand (Frost 2025).

#### Distribution

Specimens of *Kurixalus bisacculus* were found on a tree branch near a small stream between 19:45 h and 20:00 h. Males were recorded calling at the site. The surrounding habitat was a mixed secondary forest of small hardwood and shrubs.

#### Conservation

Conservation status. LC (Least Concern) (IUCN 2025).

### Theloderma lateriticum

Bain, Nguyen & Doan, 2009

ED0D1D5A-08FA-5C78-8104-B4900A364CB6

#### Materials

- **Type status:** Other material. **Occurrence:** catalogNumber: IB A.6407; individualCount: 1; sex: male; lifeStage: adult; occurrenceID: D18C5410-1428-5D83-A091-0DC3E472CECD; **Taxon:** scientificNameID: *Theloderma lateriticum*; scientificName: *Theloderma lateriticum*; class: Amphibia; order: Anura; family: Rhacophoridae; genus: Theloderma; specificEpithet: *lateriticum*; scientificNameAuthorship: Bain, Nguyen & Doan, 2009; **Location:** country: Vietnam; countryCode: VN; stateProvince: Ha Nam; county: Kim Bang; municipality: Kim Bang; locality: Kim Bang SHCA; verbatimElevation: 270 m; verbatimLatitude: 20°32.1730'N; verbatimLongitude: 105°50.3761'E; verbatimCoordinateSystem: WGS84; **Event:** eventDate: 27 April 2025; eventRemarks: collected by Nguyen QT, Pham TC, Phan QT, and Pham VA; **Record Level:** language: en; collectionCode: Amphibians; basisOfRecord: PreservedSpecimen

#### Description

**Figure 17.**
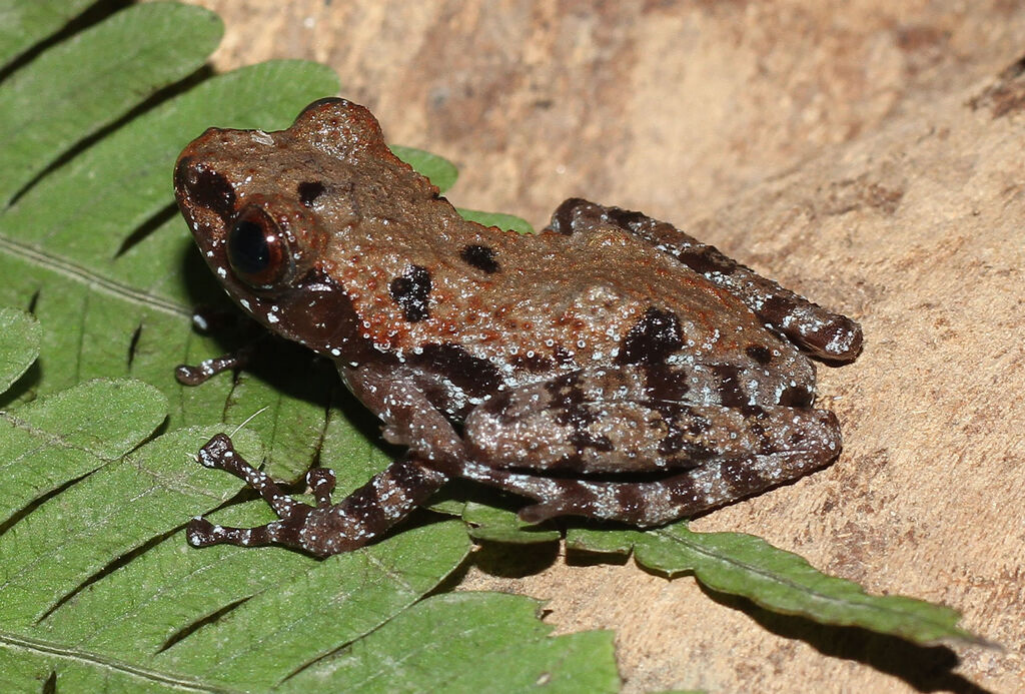
*Theloderma lateriticum* (adult male, IB A.5407).

Morphological characters of the specimen from Ha Nam Province agreed well with the descriptions of Bain et al. (2009) and Hecht et al. (2013): SVL 27.5 mm in male (n = 1); head longer than wide (HW 9.0 mm, HL 9.5 mm); snout subacuminate in dorsal view, longer than eye (RL 5.0 mm, ED 4.8 mm); interorbital distance 1.7 times broader than upper eyelid (UEW 2.5 mm, IOD 4.3 mm); tympanum distinct, round (TD 1.5 mm); vomerine teeth absent; tongue notched posteriorly; vocal sacs absent. Fore-limb slender (FL 14.5 mm); fingers free of webbing, tips of fingers expanded into large discs, relative finger lengths I < II < IV <III; nuptial pad present in male. Hind-limb slender, tibia longer than thigh (TbL 15.8 mm; TbL 14.5 mm); toe tips expanded into discs; webbing present, webbing formula: I1–1 1/2II1–2III1 3/4–3IV3–1 3/4V; inner metatarsal tubercle elongate; outer metatarsal tubercle absent; tibio-tarsal articulation reaching tip of snout. Dorsal skin granular, with keratinised spicules on small, isolated bumps; dermal fringes on the postaxial portions of the limbs absent; throat smooth, becoming coarsely tubercular towards the belly. Colouration in life: Dorsum rusty brown with three black spots on mid-dorsal, near eyelid on each side and near shoulder; lip and upper portion of limbs with small white spots; upper part of flanks with black blotches; throat, chest and belly grey-brown with cream spots (Fig. 17).

#### Distribution

In Vietnam, this species is known from Lao Cai, Son La, Ha Giang, Bac Giang, Quang Ninh and Ha Tinh Provinces (Hecht et al. 2013, Pham and Nguyen 2018, Frost 2025). Elsewhere, this species is known from China and Laos (Frost 2025).

#### Ecology

Specimen of *Theloderma lateriticum* was found on a tree branch near a small stream at 20:30 h. Tadpoles were observed inhabiting water-filled bamboo tubes. The surrounding habitat was a mixed secondary forest of small hardwood and shrubs.

#### Conservation

Conservation status. LC (Least Concern) (IUCN 2025).

## Discussion

Our new records bring the total number of amphibian species from Ha Nam Province to 19, comprising one species of Bufonidae, six species of Microhylidae, three species of Dicroglossidae, three species of Ranidae and five species of Rhacophoridae. Most of the records of the amphibian fauna in Ha Nam Province are known from Kim Bang SHCA. However, it is noted that the taxonomic assignment of some spcies have been changed, for example, *Hylarana nigrovittata* (Blyth, 1856) was re-identified as *H. annamitica* (Sherida and Stuart, 2018) (Sheridan and Stuart 2018), *Kurixalus verrucosus* (Boulenger, 1893) was re-assigned to *Kurixalus bisacculus* (Taylor, 1962) (Yu et al. 2010, Yu et al. 2017). Do et al. (2022) reported *Occidozyga martensii* (Peters, 1867) from Kim Bang SHCA, but morphological examination of the specimens collected from Kim Bang revealed another taxon, *Occidozyga lingnanica*, a recently-described species from China by Lyu et al. (2022). Our new findings not only fill knowledge gaps of the herpetofaunal diversity, but also underline the importance of the Kim Bang Species and Habitat Conservation Area in terms of biodiversity conservation in Vietnam. At present, habitat loss poses a significant threat to the local fauna of the Kim Bang forest, with amphibians appearing especially vulnerable to environmental disturbances caused by illegal logging, quarrying and forest fires. The urgent establishment of a new protected area is essential to safeguard the Critically Endangered Delacour’s Langur (*Trachypithecus delacouri*) and preserve the rich biodiversity of Kim Bang.

## Supplementary Material

XML Treatment for Microhyla butleri

XML Treatment for Microhyla heymonsi

XML Treatment for Microhyla mukhlesuri

XML Treatment for Microhyla pulchra

XML Treatment for Micryletta hekouensis

XML Treatment for Occidozyga lingnanica

XML Treatment for Hylarana annamitica

XML Treatment for Rana johnsi

XML Treatment for Kurixalus bisacculus

XML Treatment for Theloderma lateriticum
